# Stakeholders Perspectives on the Success Drivers in Ghana’s National Health Insurance Scheme – Identifying Policy Translation Issues

**DOI:** 10.15171/ijhpm.2016.133

**Published:** 2016-10-01

**Authors:** Adam Fusheini, Gordon Marnoch, Ann Marie Gray

**Affiliations:** ^1^Centre for Health Policy/MRC Health Policy Research Group, and School of Public Health, Faculty of Health Sciences, University of the Witwatersrand, Johannesburg, South Africa.; ^2^Department of Health Policy, Planning and Management, School of Public Health, University of Health and Allied Sciences, Ho, Ghana.; ^3^School of Criminology, Politics and Social Policy, Faculty of Social Sciences, University of Ulster, Jordanstown, UK.

**Keywords:** Policy Translation, National/Social Health Insurance, Success Drivers, Policy Reforms, Ghana, Implementation

## Abstract

**Background:** Ghana’s National Health Insurance Scheme (NHIS), established by an Act of Parliament (Act 650), in 2003 and since replaced by Act 852 of 2012 remains, in African terms, unprecedented in terms of growth and coverage. As a result, the scheme has received praise for its associated legal reforms, clinical audit mechanisms and for serving as a hub for knowledge sharing and learning within the context of South-South cooperation. The scheme continues to shape national health insurance thinking in Africa. While the success, especially in coverage and financial access has been highlighted by many authors, insufficient attention has been paid to critical and context-specific factors. This paper seeks to fill that gap.

**Methods:** Based on an empirical qualitative case study of stakeholders’ views on challenges and success factors in four mutual schemes (district offices) located in two regions of Ghana, the study uses the concept of policy translation to assess whether the Ghana scheme could provide useful lessons to other African and developing countries in their quest to implement social/NHISs.

**Results:** In the study, interviewees referred to both ‘hard and soft’ elements as driving the "success" of the Ghana scheme. The main ‘hard elements’ include bureaucratic and legal enforcement capacities; IT; financing; governance, administration and management; regulating membership of the scheme; and service provision and coverage capabilities. The ‘soft’ elements identified relate to: the background/context of the health insurance scheme; innovative ways of funding the NHIS, the hybrid nature of the Ghana scheme; political will, commitment by government, stakeholders and public cooperation; social structure of Ghana (solidarity); and ownership and participation.

**Conclusion:** Other developing countries can expect to translate rather than re-assemble a national health insurance programme in an incomplete and highly modified form over a period of years, amounting to a process best conceived as germination as opposed to emulation. The Ghana experience illustrates that in adopting health financing systems that function well, countries need to customise systems (policy customisation) to suit their socio-economic, political and administrative settings. Home-grown health financing systems that resonate with social values will also need to be found in the process of translation

## Background

### The Ghana National Health Insurance Scheme

Ghana introduced a National Health Insurance Scheme (NHIS) under Act 650 of 2003, which has since been amended by Act 852 of 2012. The previous cash and carry system had created barriers to healthcare access, excluding the majority of Ghanaians from healthcare, especially in rural communities.^1,2^ There were also reported cases of delays in seeking healthcare, with often grave consequences,^3-5^ as the sick, unable to afford payment avoided attending hospitals, clinics, and health centres. To date, in sub-Saharan Africa, the Ghana scheme remains the first nationwide health social protection scheme to include the rural and agricultural populations.^6^ The Holistic Assessment of the Health Sector Programme of Work 2014^7^ puts the coverage figure at 38% for 2014, translating to 10.3 million people. Other estimates put it below expectations - at about 34%.^8^ Nevertheless, the scheme has been praised for ensuring more equitable geographical provision, successfully achieving legal reforms, introducing clinical audit mechanisms, higher quality healthcare provision and for serving as a hub for knowledge sharing and learning within the context of South-South cooperation.^9-12^ It won the United Nations (WHO-UNDP) South-South Cooperation Excellence Award for 2010, in recognition of improving financial access to healthcare services^12^ and was acknowledged as an innovative and a global case study for social health insurance (SHI) at the 2011 Global Health Forum in London.^13^ The “successful” Ghana experience is considered unprecedented in sub-Saharan Africa when the impacts of insurance schemes are measured by membership or coverage.^14,15^

In recent years, the NHIS in Ghana has attracted increasing academic attention, with studies focusing on NHIS enrolment and renewal and access to the scheme,^8,16^ equity in coverage,^17,18^ quality and efficiency issues^19,20^ and fraud and abuse.^16,21,22^ Countries including Bangladesh, Benin, Cameroon, Ethiopia, the Gambia, Malaysia, and Nigeria among others have visited Ghana to study the scheme. However, measuring the success of a social protection programme such as the NHIS is not straightforward. Such programmes have often have multiple objectives and components which are likely to interact, with sometimes positive but also negative (unintended) effects. While the primary objective is usually to reduce poverty and vulnerability to shocks, they aim also to decrease inequality, for instance, in access to health and other social services. Social protection initiatives such as the NHIS can have a direct impact on lives of the poor by providing assistance to those most in need or protecting assets by insuring people against adverse shocks. But social protection can also have an indirect impact, empowering people and transforming societies and fostering participation and social inclusion.^23-25^ Starting points vary considerably as countries devote differing shares of gross domestic product (GDP) to healthcare. At 4.5% of GDP, Ghana spends a relatively high amount compared with many sub-Saharan countries^26^ and, as explained by Lagomarsino et al, countries adopting national health insurance reforms rarely conform to an established health system archetype.^27^ With regard to implementation there is an additional problem in finding common comparable indicators of progress. External factors such as the condition of the national economy will certainly impact and, as noted by Sachs,^28^ there may also be interventions outside of the health sector which can have a large effect on health outcomes such as regulation of the tobacco trade, school meal/feeding programmes and basic infrastructure improvements. Even in the context of such constraints many elements of implementation programmes pursued in one jurisdiction will typically be of relevance to the adopting country’s plans. The question asked here is what drives the “success” of the Ghana scheme and what lessons could be learned from Ghana’s experience by other countries seeking to identify the essential mechanisms needed to make progress in their own particular circumstances? To this end, the study examined the key elements of the scheme as set down by the legislation and subsequent implementation through recording the views and perceptions of stakeholders.

Given the praise and attention the scheme has attracted since implementation, the NHIS in Ghana could be considered a prime candidate for what is variously referred to as ‘policy transfer,’ ‘policy diffusion,’ ‘policy convergence’ and ‘policy translation’ - the exporting of policy ideas across countries.^29^ This study uses the concept of policy translation and, linked to the work of Stone,^30-32^ includes in the analysis discussion of ‘hard’ - legal, technological, and organisational elements of the scheme and what could be considered ‘soft’ elements - political and electoral positions, the nature of the economy and peoples’ willingness to join the NHIS.

### Policy Translation

Processes through which policies spread from one jurisdiction to another began attracting the attention of political scientists in the late 1980s. In Europe, it became clear in the 1990s that there now seemed to be a greater propensity for western governments to ‘lesson draw,’^33^ to see how policies operate in one jurisdiction, how they may be applied in another and identify what modifications may be needed to enable this. The literature developed to identify *what* could be transferred, as well as *how* transfers take place.^34^ This encompassed voluntary and involuntary transfer and defined policy transfer as a process by which knowledge of policies, administrative arrangements and ideas in one political system is used in the development of similar features in another.^35^ Stone draws attention to the key fault lines in the way this process is conceptualised in the closely related literatures on policy ‘diffusion,’ policy ‘transfer,’ policy ‘convergence,’ and policy ‘translation.’ For her, the early policy transfer literature was more concerned with the ability of importer governments to mediate their way through institutional obstacles to allow convergence.^30^ While the importing jurisdiction might employ different legal or organisational structures to the exporting jurisdiction, a relentless process of policy emulation was assumed to be under way. As she observes, the early diffusion and policy transfer literatures assumed that the process would be voluntary and constitute the means of promoting best practice based on observations of approaches that worked elsewhere.

The supposed inevitability of transfer, diffusion or convergence has been further challenged in recent years by proponents of the concept of policy translation*.* Policy translation has been referred to variously as divergence, hybridization, adaption and mutation.^30^ Reflecting the uneven passage of attempts to export or import policies between ‘donor’ and ‘recipient’ jurisdictions, this concept to some extent turns the transfer literature on its head and asks what will be needed to make things work on the ground, including the impact of application constraints.^36^ It predicts a “series of interesting, and sometimes even surprising, disturbances, which can occur in the spaces between the ‘creation,’ the ‘transmission’ and the ‘interpretation’ or ‘reception’ of policy meanings.”^37^ The emphasis on the extent to which policy is adapted by the importing jurisdiction (including vertical channels from national to sub-national levels) to fit with the local social, economic and political context seems fitting when developing countries are involved. Factors internal to a system such as the power dynamics of political interests and the socio-historical make-up of a polity can be a more powerful determinant of what is adopted than external factors.^30^ What may also be important to understand is the impact of ‘soft’ norms – ideas and concepts – which might be less susceptible to ‘hard’ institutional mechanisms grounded in law.^31,32^

We examine evidence from stakeholders in the Ghana NHIS about the implementation of the scheme in different parts of the country and discern the extent to which a system shaped to fit unique contextual circumstances means that policy translation to another context may be problematic.

## Methods

The study employed a qualitative approach to explore the NHIS policy implementation process, including the main success drivers, in two local government areas in Northern Ghana (Nanumba North and South Mutual Health Insurance Schemes - now district offices of the NHIS) and two sub-metro areas (Ashiedu Keteke and Osu Klottey Sub-Metro Mutual Health Insurance Schemes - district offices of the NHIS) in Southern Ghana. The rationale for adopting a qualitative approach is three-fold: firstly quantitative data is unreliable as evidenced by the difficulty in reaching consensus even on figures relating to the coverage of the scheme; secondly the study of the implementation and working of the NHIS does not lend itself to straightforward statistical analysis; thirdly the design and structure of the scheme means that a range of stakeholders are involved, often with overlapping responsibilities and it was, therefore, felt that the most effective way of achieving a fuller understanding of their views was through the use of in-depth semi-structured interviews. Data from these could then be used alongside other academic literature and documentary sources to enhance the validity of the analysis. As the implementation of the scheme is an evolving process involving collaboration between stakeholders, interviews needed to allow stakeholders to express a view about the schemes evolvement over time and lessons learnt.

Justification for the geographical context of the study relates to the long standing inequities in healthcare access in the country, with the South benefitting more than the North.^38,39^ The Nanumba North and South district branches of the NHIS are located in two rural districts of the Northern region. The main activities in these areas are subsistence farming and one-man businesses. Poverty levels in the districts, as per Northern region as a whole, are high and pervasive. The Osu Klottey and Ashiedu Keteke sub-metropolitan district branches of the NHIS are located within the Accra Metropolitan Assembly in the heart of Accra, the national capital. The Accra Metropolitan Assembly is urban and has better public and private health facilities as well as a large number of medical personnel. It is also better endowed resource-wise. For instance, while the Northern region had a poverty incidence rate of 50.4% in 2012/20113, in Greater Accra it was 5.6%, 18.6% lower than the national rate of 24.2%.^40^ In terms of extreme poverty, the Northern region accounts for slightly over a quarter of the extreme poor in Ghana, far more than any other region with an incidence rate of 22.8% in 2012/2013.^40^ The Ghana Shared Growth and Development Agenda 2013 affirms that on almost all socio-economic indices, rural Ghana compares unfavourably with urban Ghana with the North-South divide representing the main cleavage line in terms of poverty.^41^ At the time of the interviews (201l/2012) health insurance coverage was 82 695 out of a total population of 117 525 with an active membership of 43 901 for the Ashiedu Keteke sub-metro scheme. For the Osu Klottey sub-metro scheme the estimated active membership was 52 979 out of a total population of 121 723. The Nanumba South Mutual Health Insurance scheme had a total membership of 53 684 (both active and non-active) out of a total population of 93 646, while the Nanumba North had 86 282 registered members (both active and non-active) with an estimated active membership of 21 012^ [1]^ (as of June 2013) out of a total population of 141 584.^42,43^ Even within Ghana it was clear that a simple process of countrywide replication was not possible, given social and economic contextual differences between these two areas.

Each set of stakeholders involved in the design and implementation of the scheme has a role crucial to the effective functioning of the NHIS but also have their own actor-centred interests. Participants were purposefully selected following a review of policy documents and stakeholder mapping. A total of thirty three in-depth interviews were conducted in the four case areas over a period of eight months from June 2011 to December 2011 and again in February 2012. In the Northern region, a total of ten interviews were conducted comprising managers and former managers of the then mutual health insurance schemes, public and private health service providers, regional and former regional managers of the NHIS and a participant from a professional medical association. In the Greater Accra region of Southern Ghana, a total of seven interviews took place with scheme managers, regional and former regional managers and public health service providers. The remaining 16 interviews were conducted with national level actors and stakeholders, donor agencies and non-governmental organizations (NGOs). In this way, the views of a diverse range of stakeholders were obtained including managers of implementing agencies and institutions at regional and district levels, service providers, politicians and policy-makers, interest groups and professional associations, international donor partners and NGOs. While undoubtedly the views of users are important to a full understanding of how the scheme is functioning, obtaining an exactly representative range of user views was outside the scope of this study. This is, however, an important area for further investigation. The interviews were conducted in the English language by the lead author as part of a PhD study subsequent to appropriate training.

Interview topics centred broadly on: the principles underpinning the scheme; perceptions of the policy-making process in establishing the NHIS; political commitment to the NHIS; the structure and governance of the scheme; the financing and sustainability of the scheme; membership and entitlements and exemptions; issues relating to audit and regulation; the importance of geographical location to the successful implementation of the scheme; and participants’ views on the policy impact or outcomes of the NHIS. Interviews were recorded using a digital audio recorder and were transcribed with the assistance of software called *express scribe*. Interview data was coded based on key themes emerging from the interviews.

## Results

In this section, the ‘hard’ and ‘soft’ elements identified as critical to the success of the NHIS in Ghana are discussed.

### Hard Elements of the National Health Insurance Scheme and Policy Translation Potential

Developing countries vary considerably in their institution building and bureaucratic capacities, a factor that will be reflected in abilities to raise money, allocate resources, audit registration of subscribers, accredit providers, deliver services, and evaluate impact. The main elements of the Ghana scheme are examined below.

### Financing

Ghana funds the NHIS on a multi-source basis. In simple terms, this spreads the burden between taxation and members’ contributions. The sources comprise the national health insurance levy of 2.5% value added tax (VAT) on goods and services; 2.5% of formal sector employees contribution to Social Security and National Insurance Trust (SSNIT); parliamentary allocation to the National Health Insurance Fund (NHIF); investments made by the Authority; grants, donations, gifts and any other voluntary contributions; fees charged by the Authority in the performance of its functions; contributions made by members of the scheme; and moneys accruing under section 198 of the Insurance Act, 2006 (Act 724).^44^ While the exact funding balance is unlikely to be replicated by other countries, the principle of multi-sourcing is certainly ‘translatable’ although the Ghana system does imply a high degree of fiscal coordination, which may be hard to develop in the short-term.

### Governance, Administration, and Management

A 17 member National Health Insurance Council (NHIC) manages a National Health Insurance Fund. The Council provides subsidies to district offices of the scheme, regulates the insurance market and monitors service providers. The National Health Insurance Authority (NHIA), headed by a Chief Executive Officer (CEO) provides administrative support to the NHIC in the implementation of the scheme. To facilitate choice, the Act provides for the establishment of private sector schemes (private mutual and private commercial health insurance schemes) but these are not liable for subsidies from government and operate as insurance schemes based on a premium, contract, and policy. Private mutual schemes are not-for-profit, but private commercials are business entities and can make profit. As part of the process of monitoring the quality of services and dealing with conflicts, a health complaints committee or unit of the NHIC is situated in every district office of the scheme. With this degree of choice and complexity comes opportunity for corrupt practices even though Ghana enjoys a lower level of corruption than most developing countries.^45,46^ This is a factor that may be significant in attempts to translate the scheme elsewhere.

### Membership of the Scheme

Enrolment and membership in the NHIS is mandatory for all residents of Ghana except those working with the Ghana Armed Forces, the Ghana Police Service or those who can prove they hold a health insurance policy.^47^ The 2013 annual report of the NHIA noted the addition of three categories of membership comprising Ghana Police, Military, and Security Services. These three categories constitute 0.1%, 0.2%, and 0.003%,^48^ respectively of total membership (as of December 2013). Persons eligible for membership are expected to pay a contribution of between GHC 7.2 and GHC 48 per year (equivalent of US$7.74 and US$51.86; £4.31 and £28.76 at the time of passage of Act 650). Under Act 852, a period of 60 days or 2 months could lapse between registering and issuance of ID cards to access service. This waiting period has been reduced to one month under the new biometric card registration scheme first piloted in 2013.

Some groups are exempt from paying contributions or membership fees: children under-18 years; pregnant women; mentally ill people; indigents; categories of differently-abled persons determined by the Minister responsible for Social Welfare; pensioners of SSNIT; contributors to SSNIT; a person above 70 years of age; and other categories prescribed by the Minister.^44^ A capacity to accurately categorise the population should not be assumed to exist in all developing countries. This may be one hard element of the scheme, which is a decisive factor in policy translation.

### Service Provision

The benefit package under the scheme covers about 95%^ [2]^ of common diseases affecting Ghanaians. Service providers (public, private, or religious) have to apply to the NHIC/NHIA for accreditation and licensing to provide a specified set of services from the benefits package according to their assessed competency. Effective functioning of this aspect the scheme assumes an ability to impose licensing criteria and prevent corrupt means of acquiring accreditation. The necessary bureaucratic standards, freedom from corruption and ability to weed out under qualified providers will be hard to acquire in many other countries. It is of some significance that Ghana is perceived by experts to be one of the least corrupt countries in sub-Sahara Africa, with only Namibia and Botswana ranked better in the influential transparency international survey conducted in 2011 and in 2015 only six countries ranked better than Ghana in the Corruption Perception Index.^46^

### Technical and Organisational Elements of the Scheme

Technical, capacity, and organisational elements including the establishment of a Claims Processing Centre and the implementation of clinical audit in 2010, the establisment of the Consolidated Premium Account,^ [3]^ and the issuance of instant biometric ID cards were all identified as critical success drivers of the NHIS by research participants.

That Ghana has found it necessary to procure and utilise fairly advanced data management systems to run the scheme implies an ability to manage technology. While the need to offer such a capacity may represent an initial obstacle to adoption elsewhere, it should also be noted that Ghana is providing an implementation test bed which may ease the adoption of similar but adapted technology elsewhere. For instance, a significant development in Ghana has been the introduction of a clinical audit system in 2010. This seeks to interrogate historical claims data to identify fraud and achieve value for money in the purchasing and supplying of services. The value of this was acknowledged by a number of stakeholders including a consultant who highlighted problems in the earlier stages of implementation:

*“Many scheme managers … many healthcare providers have built houses, and many pharmacists have built houses in no time because there are too many loopholes in the system that people can tap in. I can treat you for one disease and bill you for something else that I deem fit; there is no way you would know”* (Consultant).

*“The clinical audit system is quite innovative, which helps us to uncover challenges with quality and cost”* (Director, NHIA).

*“… The health insurance Authority conducts what we called clinical audit and it finds some weaknesses and those weaknesses … are opportunities for us to take and improve the clinical care system”* (Bureaucrat, GHS).

The Authority recovered about 21 million Ghana cedis (the equivalent of £7 043 829.27/$11 188 066.22), from service providers in 2010 through the clinical audit system and helped to minimise the fraud reported in initial implementation. Such institutional and internal structural measures have enhanced the efficiency and improved the adaptability of the scheme. In addition to these measures, the establishment of a Call Centre in April, 2012 (after data collection for this study had been completed) was an effort to provide NHIS subscribers and other stakeholders with immediate advice on issues that they may have with the scheme. The technological capacity to create a Call Centre with associated administrative requirements represents another challenge for would-be importers of the scheme, but is another example of an innovation which may be viable for other countries having been developed and utilised in Ghana.

### Soft Elements of the National Health Insurance Scheme and Policy Translation Potential

A summary of stakeholders’ views regarding the soft transfer elements is presented below in relation to the factors identified.

### Background or Context of the Health Insurance Scheme

The importance of the policy context of the scheme was stressed by many of the participants. The negative experience of user fees or the cash and carry policy created the impetus for a more humane health financing system and also made mobilising social support through contributing via premiums and taxes in a pre-payment system much easier. The financial access barrier to healthcare created by the cash and carry system provided legitimacy and acceptance for the NHIS as acknowledged by stakeholders:

*“The cash and carry was just too harsh. So coming out of that and having a system like this which is in-between I think helped….”* (Deputy Director, NHIA).

*“The success factors in the cost recovery scheme were necessary for the success of the NHIS …. The fact that people were already paying for services was very important. The structures for managing resources were already there in the health facilities and … there were several things that were developed over many years of the implementation of the cost recovery or the cash and carry. That actually made it easy for us to set the NHIS”* (Bureaucrat, MoH).

*“The main issue was with the cash and carry as people were required to make deposits before medication could be given or sometimes treated … or following emergency treatment you needed to pay before medication will continue” (*Regional service provider).

### Innovative Ways of Funding the National Health Insurance Scheme and Broad Exemptions

The funding method was perceived by almost half (16 out of 33) of the interviewees across a range of stakeholder groups as innovative. There was a dominant view that a mixture of funding sources would result in greater security. The Ghana scheme rejected the classical SHI approach, where formal sector subscribers’ contributions are based on payroll deductions. This had been a source of debate during deliberations about the initial legislation and some interviewees reflected on the debates about the use of a means-test for determining how much members should contribute with key concerns relating to the adoption of such European perspectives which did not fit the Ghanaian context. A bureaucrat from the Ghana Health Service was one of those who had been critical of the means-test approach:

*“I argued that why would we do what we called book calculations and European mean-testing which do not exist here? ... For instance, if I say that 18 years and above should pay, it presupposes that once you are 18 years, you are capable of working and earning your own premium to pay and that is what ought to happen like in Europe but in Africa here, the truth on the ground is that even though the amount is small per head, the payment is paid mostly by heads of families”* (Bureaucrat, GHS).

In the end the legislation resulted in contributions coming from a special and guaranteed fund at SSNIT on the grounds that this would increase the likelihood of the most excluded and vulnerable populations being covered. A number of scheme managers argued that the policy is not target-specific and sufficiently focused to ensure that those who really need services (particularly the poorest and most disadvantaged) get them. A Development Partner expressed the view that ‘*Creating blanket access to so many people’ created problems.’*

However, comments about the funding model among NHIA officials were broadly positive as reflected below:

*“What is very innovative about Ghana’s health insurance is that unlike other African countries, they talk of SHI and they look at the classical SHI where it is based on payroll deductions and so if you are not earning something on the payroll, you are out of the system. That is not the case here”* (Deputy Director, NHIA).

*“No African country has been able to copy our system because this is a tax-financed programme …. Many of the African governments say ‘Oh as for us politically, we cannot do this tax’ … if you cannot do the tax, how are you going to finance a social programme like health insurance?”* (Former CEO, NHIA).

### Hybrid Nature of the Ghana Scheme

The term hybrid is used to denote the use of existing health system elements where appropriate. It was suggested that Ghana’s adaption of already existing community-based mutual health organisations ensured a quick scale up in coverage. The significance of this was noted by a number of participants as exemplified by the quotations below:

*“When the issue of health insurance got to the agenda of government, the idea was to use the CBHIS structures to develop the new healthcare financing system in Ghana which is the current NHIS that we have”* (Development Partner).

*“By the time that this thing (NHIS) was happening, about 57 districts had community health insurance schemes but they were all doing Out Patients Departments (OPDs). So you can now say from that point, there was something that informed the then coming government that look, after all there is something that is working, we can do it on a national scale”* (Bureaucrat, GHS).

*“Ghana built a hybrid system as [it] had community-based health insurance schemes, which comprised people like you and I, who had voluntarily come to agree that we want to pool our risk together so that in the case of any incident, we support each other from the common basket”* (NHIA Official).

Both the formal and informal sectors were combined in one national system. In other developing countries, the tendency is to first cover formal sector employees, which affects the pace of scale up of coverage given the large informal sectors in most African and other developing countries. The potential to translate the Ghana NHIS might depend on prior financing systems being in place in a host country. However, caution must be urged with respect to the possibility of integrating existing institutions such as the community-based insurance schemes in a national system as Ghana did.

### Political Will, Commitment by Government and Other Stakeholders and Public Cooperation

A cross section of interview participants spoke of how political will has been a key success driver of the NHIS. Since implementation, a bi-partisan approach has been adopted to national health insurance issues. That all political sides support the scheme for most of the time, should be considered a major factor in successful implementation:

*“When you talk about the reasons for the success, it is the political will. ….it has been non-partisan. You talk to whichever divide of the political system; they all say health insurance is very good to us”* (Director NHIA).

*“The government’s determination to make it work is very important and so they provide as much support as possible”* (Donor Partner).

*“The interest government has in the operations of the scheme has helped to make a very important headway because there is that political will to ensure the implementation of the policy”* (District Scheme Manager).

Political support for a collectivist version of National Health Insurance (NHI) implied an electoral mandate for this innovation but it also needed support from other stakeholders. Prior to and during implementation, NGOs and international bilateral and multilateral development partners such as the Danish Agency for International Development (DANIDA) and others, engaged in education and public awareness concerning the creation of the NHIS. This sensitisation led to public acceptability and strong support for the programme within Ghanaian society:

*“What I am saying is that people accept it both at the grassroots level and at every level of our society… and that is one of the key ingredients to our success”* (Director NHIA).

A former Health Minister summed up the importance of support by the people in the following words:* “I still think that if you don’t get the kind of people we have here and they cooperate, I think we cannot move forward.”*

A participant from the Trade Union Congress (TUC) noted that while the organisation had opposed what it saw as the lack of engagement around the legislation for the NHIS the idea has broad public support:

*“We moved round the country to talk to working people and there were some levels of apprehensions … but all agreed that it was worth introducing NHI”* (TUC).

A Regional Service Provider spoke of the feeling that the NHIS would address historic inequities:

*“…The NHIS was going to bring some sanity because people can access healthcare; we were also going to get money at least at the end of it. This thing of I cannot pay was going to be a thing of the past and as members of the GHS we were going to implement it”* (Regional Service Provider).

In the context of policy translation, the existence and strength of cross party and cross community political support in host countries is a significant factor but one which will vary from country to country. Prior to the NHIS, cross party agreement on substantive policy issues in Ghana was rare and interestingly, while it appears to be a strong factor in implementation of the NHIS, it is not a feature of contemporary Ghanaian politics in other policy areas.

### Social Structure of Ghana (Solidarity)

Strong social bonds (solidarity) underpin the success of SHI systems all over the world and the Ghanaian case is no exception. Ghana has been successful in integrating traditional social kinship ideas into the modern NHIS system. This was emphasized during the interviews:

*“We are pro-poor and looking at our social system- the extended family system, where we are each other’s keeper, health insurance has been a relief for the common person with the card. Because you just walk to the hospital; you get your care and then walk back home. That is the difference it has made”* (Christian Health Association of Ghana).

*“Health insurance in my view and principle is a good thing. It demonstrates solidarity one can show to others like those who are capable now paying for those who are unable. Also it’s like a pool, you pool resources together to ensure that both those who can afford and cannot afford are all given some opportunities to secure medical care”* (TUC).

A former regional manager also observed that principles underlying health insurance including the risk pooling and redistributive element are synonymous with traditional Ghanaian mechanisms of social support:

*“The social structure of Ghana is the basic advantage that we had from which the programme sprang because already existing in tradition is self-help spirit. So, the collective will of the people of Ghana to ensure that certain programmes succeed is the underlying factor”* (Former Regional Manager).

Principles underpinning the NHIS ensure it resonates with the citizenry as it is in line with certain established societal values, norms and customs. Many participants likened the health insurance to the “Nnoboa”^ [4]^ concept associated with the Akans^ [5]^ and other groups. It is a communal process, where members of a community come together in a show of solidarity to support each other for the benefit of the entire community. The concept of insurance translates to this concept through the pooling of funds and spreading of risks so is not entirely new or alien to Ghanaians.

### Ownership and Participation

Structures and institutions for accountability and transparency were created during the initial implementation of the scheme. At the district level, various structures including district health insurance communities and health insurance general assemblies were established. These created platforms for community ownership and mobilisation of public support was consequently a less difficult task. This conclusion was affirmed strongly in the interviews:

*“We also made the district schemes accountable. Management at that level were held accountable by the people in the community because community health insurance committees were set up to help do the mobilisation of the people into the scheme, help do the collection, and help distribute ID cards”* (Deputy Director, NHIA).

*“If you decentralise something and say the community owns that thing and that you have control over whoever is the manager in there, they are proud of that. This is our scheme and so we will pay and manage it”* (Senior Research Officer, NHIA).

*“The district scheme concept was adopted and that was one thing that also rapidly increased enrolment … you see, it picked up because it was decentralised and the people were competing among themselves to get people enrolled”* (Bureaucrat, GHS).

A decentralised approach seems to have created a sense of belongingness, trust and confidence. However, the amended Act (Act 852) has taken the ownership away from communities by making the DMHISs district branches or offices of the NHIS; arguably they are no longer channels for community participation. It is likely that ownership and its mobilisation as an implementation force would be significant in policy translation in other African countries. The concept of ownership is context-laden and needs to be considered on a country or even regional basis within countries.

### The Importance of Area Differences

While participants identified what could be considered soft or hard transfer success drivers, the majority also noted the impact of regional differences on the success of the scheme. For instance, there was reference to how the pervasive poverty in the North impacts on the finances of schemes in the area. Subscribers in Northern and Southern Ghana pay between 7.20-12 GHS (equivalent of US$1.66-2.77) and between 20-35 GHS (US$4.61-8.06) per annum, respectively, in the low-income socio-economic groups. Though this is a relatively small amount, the culture in the North, where the head of the family bears all the responsibility, means it becomes a problem. This was highlighted by one of the respondents:

*“The contribution is paid mostly by heads of families. Particularly, when you go to the North, they are farmers in the communities, … but … you cannot sell all your animals just to pay for premiums and then none of them is sick, that sort of thing, and so the family head begins to think … the premium he has to pay alone in a year, he knows that if he has to do that he has to dispense of some animals, he has to dispense of some food and … so that also becomes our big problem” (Bureaucrat, GHS).*

Spatial distribution of health facilities and personnel was also identified as a major factor impacting on successful implementation in the case study areas. There is evidence of skewed distribution of facilities and health personnel in favour of the South. In 2009, the Greater Accra region had 466 health facilities while the Northern region had 300; the Greater Accra region had 4662 hospital beds with 1295 in the Northern region. The doctor to population ratio was 1:5103 in Greater Accra to the Northern region’s 1:50 751 in 2009. A political actor and key government official stressed the uneven health facilities and personnel distribution between the North and South and described how this could be a challenge for policy translation:

*“You need to have facilities; you need to have human resources. The health facilities should be spread across the country, sometimes access to the place should be enhanced … If I have a card, I run to a health centre, the doctor is there but the theatre equipment is not working, and I need theatre service … the card is as useless as if I did not have”* (Politician).

In the Northern region, certain areas or communities have no access to even Community-based Health Planning Services (CHPS), the basic primary healthcare facilities. The accessibility problem is exacerbated by poor road infrastructure, especially in the rural areas of the North and travelling from rural areas to access healthcare is mostly done by walking or by bicycles with communities and health facilities sparsely located. Although road infrastructure is better in the South, vehicular traffic and road congestion in big cities like Accra and other urban centres makes travelling difficult. Thus, while in most parts of Europe or America travelling a distance of 24 km would take a relatively short time; in Ghana it could take 2 to 3 hours presenting a serious hindrance to the implementation of the health insurance programme. In the Northern region, the issue of hard-to-reach communities was described as a critical problem affecting successful implementation:

*“Sometimes there are some communities or facilities that are overseas (across a river where you have to go by using a canoe). So in order to get to these people, particularly, during a particular period (rainy season) within the year, you have to go through a hell of time”* (District Scheme Manager).

## Discussion

Ghana’s system may be viewed as somewhat unique. Unlike in other developing and middle-income countries, the funding approach ensures coverage for all those who subscribe to the scheme. This model may not transfer easily to other African countries where the required degree of fiscal discipline is not in evidence. Even Ghana, regarded as a country capable of using centrally located financial levers to good effect, found that regional socio-economic differences impacted on the implementation of the NHIS. In many other sub-Saharan African countries, there is little record of government committing to collect taxes on a scale required by a NHIS and far less evidence of the organisational capability required to collect revenues in a timely and regular manner. Public expenditure management and the ability to maintain a consistent budget process is often difficult to achieve in African countries.^49^ This finding is consistent with the observation that the success of social protection programmes such as the NHIS is down to the ability of policy managers to innovatively find a variety of ways of financing, including domestic sources, aid from international donors and private or NGO financing sources.^23^

Stone observes that the extent to which importing jurisdictions adapt policies to fit with the local social, economic and political context is crucial for the success of such policies.^30^ This observation is apparent in the Ghanaian case with the adoption of a funding strategy and mechanisms built on a combination of classical SHI ideas but utilising the community-based mutual health organisations that existed in Ghana. Ghana has been able to avoid compromising the implementation of NHIS through path dependency.^50-52^ Path dependency theory emphasises that institutional history matters in understanding how new problems are encountered and why strategic possibilities are identified, missed, rejected or adopted and must be considered as an important factor in prescribing the fate of policy translation to other African countries. Stated simply, there may be obstacles rather than help present in the form of existing organisations and institutional practices. For instance, even in the case of Ghana, there was some resistance from the community-based insurance schemes seeking to maintain their identity. In countries such as South Africa, with a well-resourced private sector and extensive medical aid schemes, incorporating these into a NHIS could be challenging. Thus, designing a scheme with “a rich blend of the traditional and modern”^5^ appealed to people. Clearly this is a highly contextualised form of implementation support. This situation is not unique to Ghana and similar examples of traditional social solidarity will be evident in other African countries. This is an implementation resource of some significance. In countries such as Tanzania, Nigeria, Uganda, and Kenya which have adopted the classical SHI approach the result has been more fragmented schemes. This also affects the quick scale up of coverage, as those in formal employment and their families have to be covered first before it is extended to those in the informal sector when resources become available.

In the case of Ghana, there was clear support from the political party (NPP) and the then President who wanted to show the electorate that his party had fulfilled an election pledge.^53^ Although the main opposition party (NDC) was opposed to the NPP’s approach due to dissimilar political agendas and ideological convictions, the fact that it was a decentralised programme and provided a platform for community participation and ownership of the schemes meant that they accepted the basic taxation model used to fund the NHIS. This degree of social solidarity may not be evident in other African countries due to fear of political backlash for instituting new or increasing taxes.

The scheme in Ghana also had the support of professional associations such as the Ghana Medical Association and the TUC who wanted an alternative to the cash and carry system and a mechanism that would improve healthcare delivery in the country. As established in the study, solidarity is crucial for the success of health insurance systems and Ghana has been able to achieve with support from different stakeholders. This finding conforms to the study of Fenenga et al^54^ in the Greater Accra and Western regions, where the significance of community solidarity and social networks in supporting the NHIS has been identified. In other African countries such as South Africa, where private practice is quite strong and well-resourced, the medical profession may not provide the same degree of support fearing damage to their earning power. The findings of this study endorse those of Basaza et al who argue that there needs to be greater attention and focus on recognising, analysing, and managing the political interests of key stakeholders during the policy process.^53^

The findings are in keeping with the position that policy translation entails a “series of interesting, and sometimes even surprising, disturbances, which can occur in the spaces between the ‘creation,’ the ‘transmission’ and the ‘interpretation’ or ‘reception’ of policy meanings.”^37^ It should be noted that developing the institutions of clinical audit and accredition along with the establishment of the Call Centre have refined the Ghanaian situation, so what is translatable is not static. That Ghana has the administrative, organisational and technical capacity to establish such institutions confirms findings by Jehu-Appiah et al, suggesting that in addition to price and benefits, convenience as facilitated by technical capacity, was a significant scheme factor associated with enrolment and retention.^55^ Such capacity also implies that Ghana has been able to deal with the initial implementation challenges of gaming, fraud and abuse of the system, which might not be easy for importing jurisdictions. This is critical; as has been noted in a joint study carried out by the World Bank and Harvard University the Kenyan experience of SHI implementation included instances of officials diverting large sums of money derived from the surpluses in insurance funds into their own bank accounts.^45^ Witter and Garshong^56^ view the sustainability of NHIS as problematic unless means are found to increase the cost-effectiveness of purchasing and the responsiveness of the system as a whole.

Our findings also concur with those of Giovannetti et al with regards to conditions or factors for success of the scheme, which they identified as including fiscal sustainability, administrative capacity, and political commitment.^23^ There is no doubt that there are significant challenges to policy translation. The Ghana approach defies neo-liberal propositions concerning how health services should be financed. Contributions from the informal sector are not actuarially calculated, neither are formal sector contributions rated based on healthcare costs. The question remains as to whether such a political economy is translatable in other economies.

## Conclusion and Implications

The study examined the drivers identified by stakeholders as being crucial to the success of the Ghana scheme. What then is relevant for other countries? There are three clear findings emerging from the study. In the first place, health-financing systems in low- and middle-income countries ought to rely on a mix of funding sources given the structure of the economies and labour market. A prevalence of low taxation capacities and large informal sectors imply no one source can generate enough revenue to provide health services for the whole population.^57^ Thus, combining innovative and multiple sources of financing healthcare may be more feasible for developing and middle-income countries. Secondly, solidarity, a key principle of SHI, must be produced “organically” from existing sources or by the political elite if not present within the society. Solidarity encourages cross subsidisation and risk equalisation. Without solidarity, progress would be curtailed because the success of SHI and indeed any health financing system depends to a large extent on the size of the pool of willing contributing members. Thirdly, history and context matter in designing health financing systems. In Ghana, the harsh realities of cost recovery with stories of how people died because they could not afford hospital fees served as reminders and helped maintain and sustain the system.^58^

Given the extent to which stakeholders referred to specific features of Ghanaian society in explaining how the programme had been implemented, it is concluded that other developing countries can expect to translate rather than re-assemble a national health insurance programme. This is likely to be in an incomplete and highly modified form over a period of years. The process may best be conceived of as germination rather than emulation. The Ghana experience illustrates that in adopting health financing systems that function well, countries need to customise systems to suit their socio-economic, political and administrative settings. Home-grown health financing systems that resonate with social values will need to be found in the process of translation.

There are also unanswered questions when it comes to assessing the prospects of translating Ghana’s NHIS in other African countries. Modelling the implementation of a complex programme is hampered by the inherent difficulties in weighting different elements of the implementation field with respect to their influence on outcomes. The current study has demonstrated this problem by examining stakeholders’ knowledge of the implementation process. For example, in an African context Ghana in implementing NHIS may be demonstrating an elevated capacity to apply effective governance practices to health services. The Ghanaian scheme, by African standards, relies on a fairly sophisticated system for raising taxes and coordinating public spending programmes. At an operational level of governance there is a considerable analytic capacity employed in clinical audit. Clinical audit as well as providing a basis to ensure quality and safety standards, has also been proven to be useful in identifying cases of fraud. Implementation may also depend on the inclination of the political class acknowledging problems and finding ways through a problem rather than ignoring it.

Would the existence or absence of one of these attributes prove decisive in implementation in another African country? Is it possible that other countries may have different but equally effective governance capacities in helping secure the implementation of a scheme similar to NHIS? Is it possible that failings or problems with governance can be compensated by strengths elsewhere? The study identified the significance of regional differences in the implementation of the scheme. Ghana has a physical geography that tends to render regional differences in wealth, clinical facilities and staff more significant than they would be in a country where travel was easier. Spatially related disparities will be evident in most African countries but perhaps to a lesser extent in some cases. More qualitative case studies are needed to provide the context rich data that policy analysts require to make sense of the relative significance of factors influencing implementation.

## Limitations of the Study

We acknowledge that the findings of the study cannot be generalised beyond the four case study areas as the study covered two district offices of each the NHIS each in the Greater Accra and Northern regions of southern and northern Ghana, respectively. However, findings can serve as pointers to other countries seeking to replicate Ghana’s experience. Finally, the study covered the implementation process from 2004-2013 but it is acknowledged that policy and implementation processes are complex and can be affected by both endogenous and exogenous factors.

## Acknowledgments

The research was made possible by the award of University of Ulster Studentship to Adam Fusheini.

## Ethical issues

The study received ethical approval from the Research Ethics Committee at Ulster University in Northern Ireland, UK and from the Ghana Health Service. Permission was also obtained from the NHIA to visit the then selected District Mutual Health Insurance Schemes (DMHISs). Participants provided written consent of their willingness to take part in interviews and for these to be audio recorded. All identifying features of participants were kept separate from the main data in order not to breach guarantees with respect to anonymity.

## Competing interests

Authors declare that they have no competing interests.

## Authors’ contributions

Research design, interpretation of results and writing were shared equally among authors. AF conducted fieldwork in Ghana and analysis of data. All authors also read and approved the final manuscript for submission.

## Authors’ affiliations

^1^Centre for Health Policy/MRC Health Policy Research Group, and School of Public Health, Faculty of Health Sciences, University of the Witwatersrand, Johannesburg, South Africa. ^2^Department of Health Policy, Planning and Management, School of Public Health, University of Health and Allied Sciences, Ho, Ghana. ^3^School of Criminology, Politics and Social Policy, Faculty of Social Sciences, University of Ulster, Jordanstown, UK.

## Endnotes


[1] Updated by the MIS officer of the Scheme during my trip to Ghana in May 2013.

[2] NHIS benefits package: http://www.nhis.gov.gh/?CategoryID=158&ArticleID=120. Last accessed on August 05, 2013.

[3] An account where contributions from informal sector employees are paid into by the various district branches of the NHIS.

[4] From the Twi language, which means solidarity, where people come together for their self-interest and benefit through some form of support for each other.

[5] Akan is the largest ethnic group in Ghana comprising the Asantes, the Fantes, the Bono, the Akyems, the Nzema, Akwapems, etc.


## Key messages

**Implications for policy makers**

- The Ghana case demonstrates that policy translation should focus on customisation by tailoring policy ideas to fit the local contextual conditions of importing countries in order to make them acceptable, legitimate, and workable.
- The long-term viability of health insurance systems in developing and middle-income countries depends in political terms on the existence of a non-partisan approach to health financing systems. This will ensure trust, confidence, and the generation of solidarity among all stakeholders. We accept this is difficult to achieve.
- In moving towards universal health coverage, policy-makers in low- and middle-income countries need to consider multiple funding sources as a way of dealing with challenges presented by the structure of their economies and labour markets, low taxation capacities and large informal sectors.
- For policy translation to make the desired impact, policy-makers must take into account capacity constraints and opportunities for improvement within the importing jurisdiction.

**Implications for public**

The study demonstrates that while governments try to provide available healthcare through implementation of various financing mechanisms, there is a significant dependency on social solidarity. The public need to be willing to participate in National Health Insurance Schemes (NHISs) to ensure cross subsidization and risk equalization.
